# Supplementary material for: RNA Control of HIV-1 Particle Size Polydispersity
Source: PLoS One. 2014 Jan 24;9(1):e83874. doi: 10.1371/journal.pone.0083874 (PMC3901647; doi:10.1371/journal.pone.0083874)
Supplement: File S1 — Figure S1. Figure S2. Figure S3. Figure S4. Figure S5. Methods S1. (PDF) [file pone.0083874.s001.pdf]

Supplemental material

Rna control of HIV-1 size polydispersity

1 Optimization of filter parameters in order to extract VLP by automated AFM image analysis

The optimal choice of the area range appears to be important in order to focus on the VLP population. Indeed a loose preliminary selection of particles shows that the lateral dimensions of single objects for VLP purifications range roughly between 50nm to 180nm in diameter (see figure S1). This large width of the size distribution indicates the likely presence of different type of particles in the solution prior to surface deposition. Indeed, the current purification scheme based on sucrose gradient is efficient in order to collect VLPs, but it has the side-effect of letting through small vesicles of similar density called "exosomes". These are natural secretion products of cells under the present growth conditions. We quantified the size distribution of these additional particles using transfection assays with plasmids not coding for Gag or Gag-related proteins ("Mock" purifications). Since the majority of these particles have sizes smaller than 90nm (figure S1) and since VLP are not expected in this range, it is possible to get rid of the statistical contribution of exosomes within VLP purification conditions by imposing a lower size threshold of 90nm (the equivalent area is about $7000nm^2$), as it is shown in figure S1.

The different populations after low or high area selection are shown in the following table for the three conditions *Mock, VLP*$-\psi$*, and VLP*$+\psi$:

Number of selected particles	Mock	VLP$-\psi$	VLP$+\psi$
Low area min threshold	121	411	513
High area min threshold	20	211	248

In this table, *low area min threshold* and *high area min threshold* are respectively associated to different values of the lower bound for area selection.

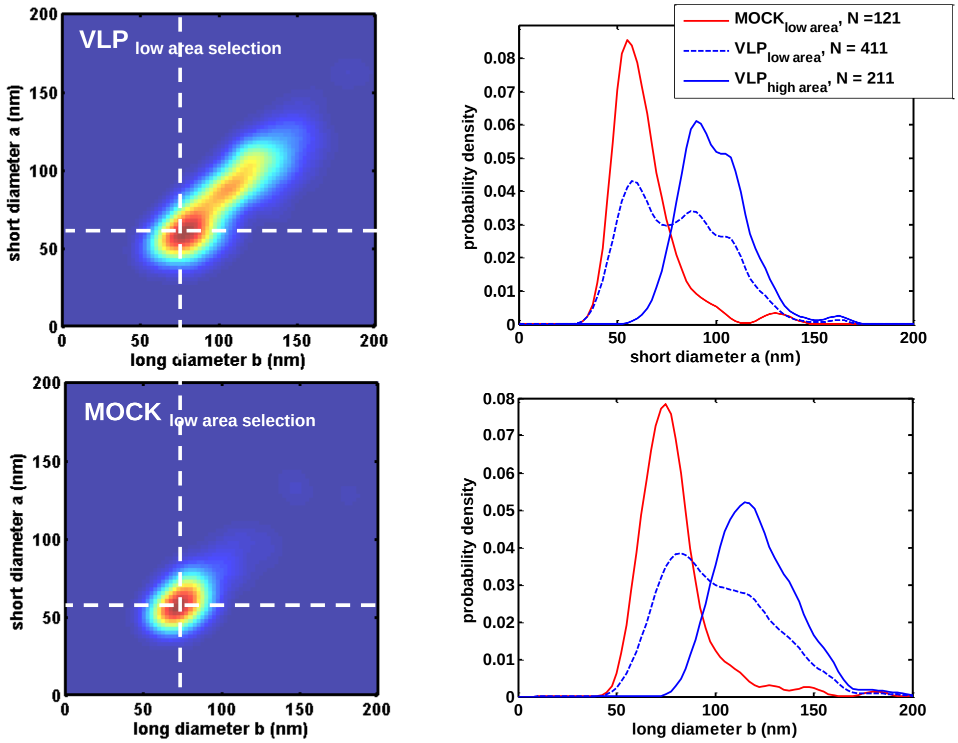

Figure S 1: Size distribution for different allowed range areas for Mock and VLP$-\psi$ particles. The loosest area selection on VLP , termed *low area selection* ($3000nm^2$ <area< 10^5nm^2), shows a large bimodal size distribution. Similar analysis on Mock purifications shows that one of the two mode of the size distribution is identical. By using higher range of area in order to analyze VLP purification (*high area selection* $7000nm^2$ <area< 10^5nm^2), the "Mock" mode is eliminated, and focus is made on VLPs. Note that the difference between low and high area selection lies in the value of the lower bound of area selection.

The larger bound for area selection is identical for both conditions. This table calls for some comments. First, the high area selection is efficient at discarding exosomes (Mock conditions), since only 16% of these particles

survives the selection. Second, the high area criterion selects roughly 50% of the initial population of both types of VLP. Most importantly, despite the apparent strong selection of VLP, the RNA control of HIV-1 size polydispersity is still observed under the low area selection. This is illustrated in figure S2. The main reason is that this effect is expected to be mainly seen on the large area side of the size distributions.

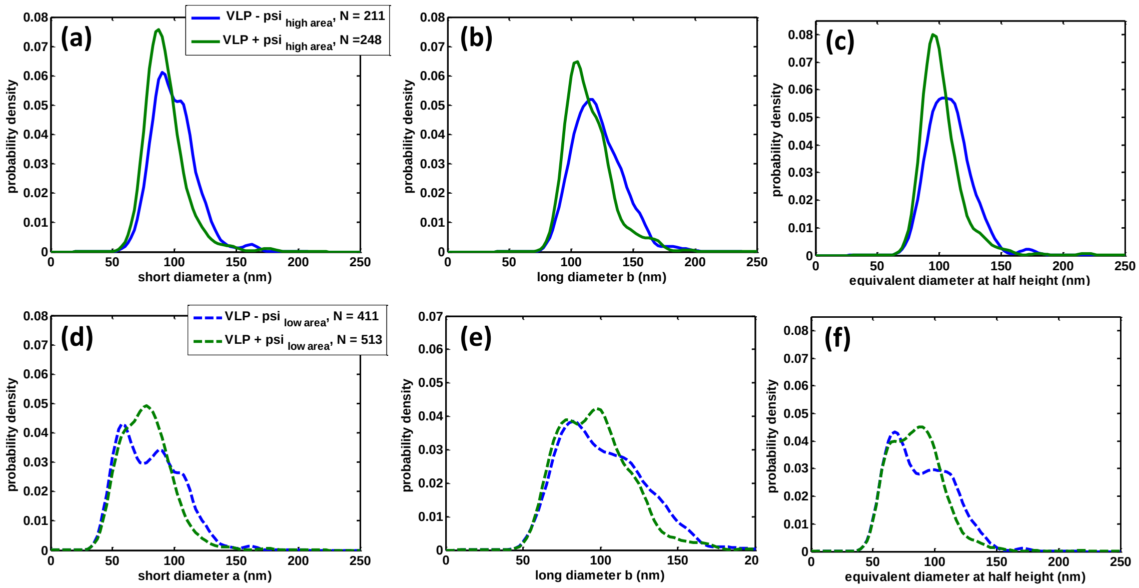

Figure S 2: Size distributions for different allowed range areas for VLP$-\psi$ and VLP $+\psi$ particles. The size distributions of short diameter, long diameter and equivalent diameter are shown for high area selection (upper row, respectively *(a),(b),(c)*) and low area selection (lower row, respectively *(d),(e),(f)*)

2 Comparison between VLP and cores

In the figure S3, the 2D histogram *minor axis/ major axis* of VLP and cores are compared. Two alternative representations are proposed: raw data for minor and major axis, and 2D histogram for the same data.

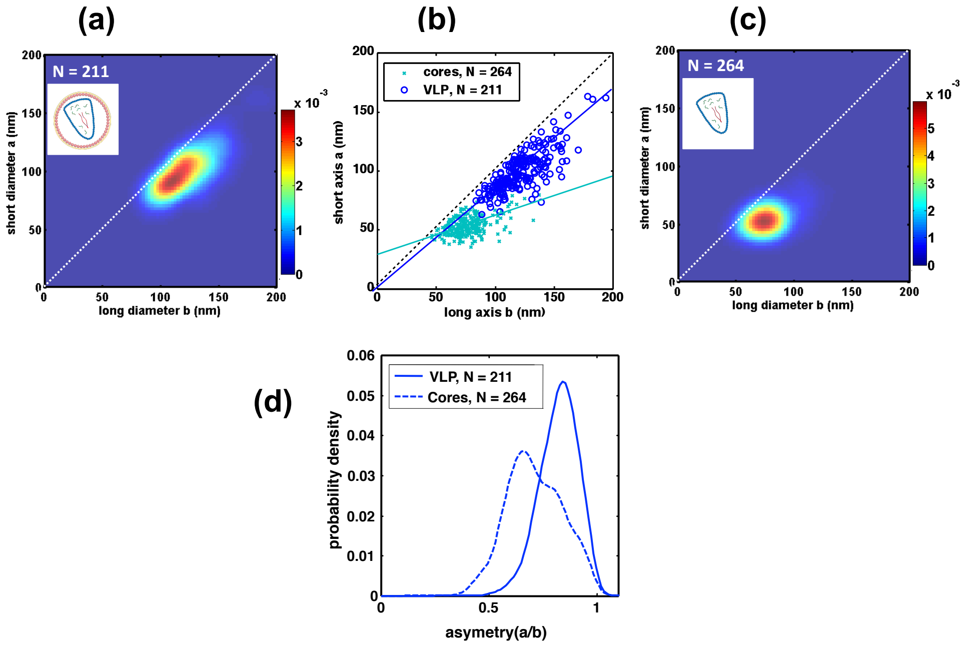

Figure S 3: Comparison between VLP and cores. (a) and (c) are the 2D histogram using a color map in order to show the frequency of events. (b) Raw data that have been used to produce the 2D histogram (a) and (c). The lines show the main direction of size fluctuations: VLP is rather isotropic, unlike cores. (d) Histogram of asymmetry a/b for VLP and cores. The position of the peak in asymmetry is shifted between VLP and cores, as expected from simple visualization of individual images.

3 Titration of Ψ-RNA

In order to check whether the amount of Ψ-RNA transfected influence the modulation of size distribution, we used three different conditions of transfection. The data presented in the main text of this work were obtained by adding 500 μl of phosphate buffer (HBS2X) to 8 μg of virus expressing plasmids, and 4 μg of a plasmid expressing Ψ-RNA previously supplemented with 500 μl CaCl2 (250 mM). This sample is labeled $+0.5\Psi$, in reference with the ratio of RNA to protein ratio. Within the new conditions described here, the amount of virus expressing plasmids is unchanged, but the amount of added plasmid expressing Ψ-RNA is now either 8 μg ($+1\Psi$) or 16 μg ($+2\Psi$). The histogram of the size distribution shown in figure S4 shows that the size distributions are statistically undistinguishable for $+0.5, +1, +2\,\Psi$. This shows that the effect of size modulation is not affected as function of viral RNA amount.

4 AFM liquid imaging of VLPs and automated image analysis

In order to confirm that our results are not dependent on the use of AFM imaging in air, we performed AFM imaging in liquid environment. In this case, AFM imaging on wet samples is much harder to achieve mainly because the biological objects are weakly adsorbed under such conditions in order to prevent VLP deformation due to strong interaction with the substrate or the use of biochemical glue (such as glutaraldehyde) that may alter the VLP conformation. Typically, with dried samples, there are between 3 to 8 particles per image (3 microns x 3 microns), while upon imaging in liquid most images we obtained do not show any particles. Moreover, VLPs tend to desorb from the functionalized mica surface after few tens of minutes when immerged in buffer in the AFM liquid cell. The observation of a single particle becomes an extremely rare event as compared to the imaging of dried samples. In addition the image quality is reduced and quantitative image analysis as we performed in air become somewhat noisy.

Technically, for AFM liquid imaging, purified VLPs in physiological TNE buffer (Tris 10mM, EDTA 1mM, NaCl 100mM, pH=7.4) were deposited on poly-L-lysine functionalized mica surface for 2 to 5 minutes, rinsed with $1ml$ of TNE buffer and then immersed in AFM liquid cell containing about $50\mu l$ of TNE buffer. Imaging was performed in Peak Force Mode with Multimode 8 AFM (Bruker AXS Inc.) in fluid using NPS or fluid scan asyst cantilevers

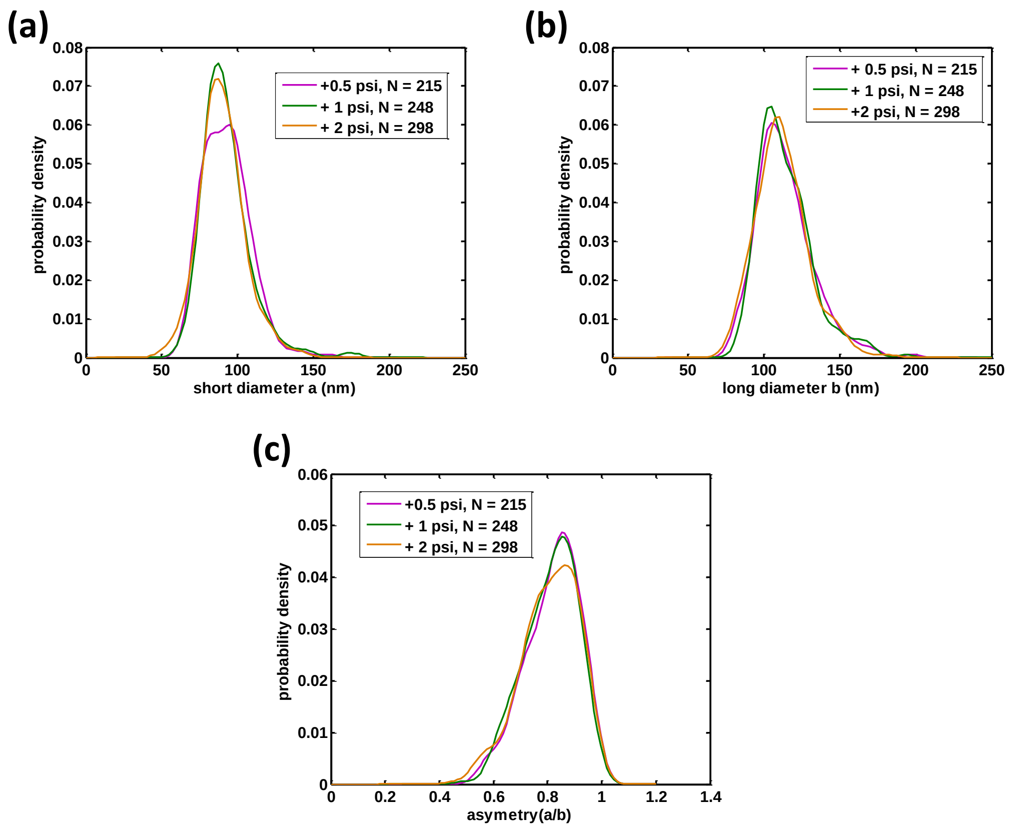

Figure S 4: Titration of Ψ-RNA on the size distribution. The label for each titration is explained in the text of supplemental data. (a) Histogram of short diameter for different titration. (b) Histogram of long diameter for different titration. (c) Histogram of asymmetry for different titration.

(Bruker). The 512 by 512 pixel image was recorded at a scan rate of 1.8 Hz. The obtained AFM images (Figure S5a) were analyzed using the same Matlab script as AFM images previously obtained in air, using the same height and area threshold selection parameters.

The presence or absence of viral RNA produces qualitatively the same modulation of size distribution as the one observed for dried samples: a larger average VLP size as well as larger polydispersity is observed when viral psi RNA is absent (Figure S5b). This is not unexpected. Indeed,

the drying process of particles may modify the absolute values of sizes as compared to liquid environment, but not the relative size difference. One of the main reason for this absolute difference in size is associated to the choice of cantilever. The AFM cantilever used for liquid imaging are necessarily different than than the one used in air and as a consequence AFM tip radius is larger in liquid (10 to 20 nm) than in air (5 nm). As a consequence, tip convolution is expected to be higher in liquid imaging leading to a slightly larger average particle diameter.

5 Models of self-assembly

We consider in this section the equilibrium properties of the capsid self-assembly. In order to describe this process, we use the standard formalism of micellization thermodynamics, which has been used extensively in order to describe the aggregation properties of surfactant solutions. The aim of this approach is to predict what will be the equilibrium size distribution c_p of aggregates of p molecules given the total initial amount of molecules that have been put in the solution. The scenario of interest for retrovirus self-assembly involves at least three distinct molecular species: Gag proteins, viral RNA and cellular RNA. Before investigating the behavior of such a complicated system, we present below the simplest case of single protein self-assembly in the absence of RNA. This will allow to highlighting the relative contribution of entropy and enthalpy to the size distribution of aggregates.

Within the simplest level of modelization of capsid self-assembly, we first consider a solution of proteins of initial concentration ϕ_0 that tend to form aggregates or capsids made of p proteins. The gain in free energy for the formation of one aggregate of size p is kTF_p, where k is the Boltzmann constant and T the temperature of the system. The Gibbs free energy of the solution of proteins is written as

$$\frac{G}{VkT} = c_1(\ln(c_1 v_0) - 1 + F_1) + \sum_{p=2}^{\infty} c_p(\ln(c_p v_0) - 1 + F_p) \qquad (1)$$

where c_1 is the concentration of free proteins, c_p is the concentration of aggregates of size p, V is the volume of the solution, and v_0 the typical volume associated to a water molecules. As a consequence, the concentration in all our calculations are expressed in units of v_0^{-1}. For each aggregate type, there is a translational entropy term $kTVc_p(\ln(c_p v_0) - 1)$ and an enthalpic term for the formation of aggregate $kTVc_pF_p$. As it is described below, this

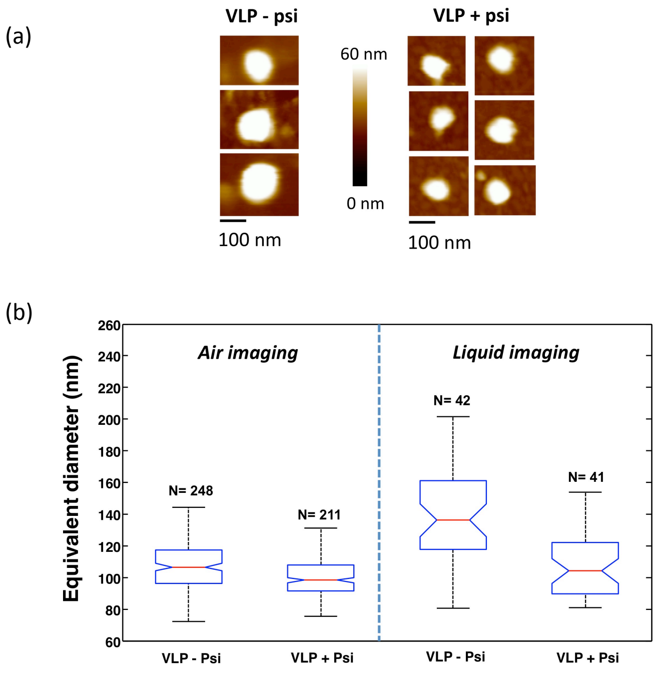

Figure S 5: *(a)* Example of VLPs imaged in physiological TNE buffer, in the absence or presence of ψ-RNA. *(b)* Distribution of VLP equivalent half height diameter represented as a boxplot where the central red mark represents the median of the distribution, and the box contains 50% of the total population of selected VLPs (the edges of the box are the 25th and 75th percentiles). The central notch on each box represents the comparison interval for the median value. Indeed, two medians are significantly different at the 5% significance level if their intervals do not overlap. The distribution are compared when VLPs are produced in the presence or absence of ψ-RNA and when the VLPs are imaged in air or liquid environment. The total of VLPs is also mentioned. Note that the statistical sampling is significantly lower when VLPs are imaged in liquid, but both effect are still present: a lower average diameter value together with a lower size polydispersity for VLPs produced in the presence of ψ-RNA.

is the balance between these entropic and enthalpic contributions that sets the precise size distribution.

This Gibbs free energy assumes implicitly that long-range interactions between aggregates are negligible. At equilibrium, the size distribution c_p minimizes the Gibbs free energy with the global constraint of mass conservation

$$\phi_0 = c_1 + \sum_{p=2}^{\infty} p c_p \tag{2}$$

This can be taken into account by the use of a Lagrange multiplier μ that is interpreted as the chemical potential of individual proteins. The equilibrium conditions are written as

$$c_p v_0 = (c_1 v_0)^p \, e^{-(F_p - pF_1)} \tag{3}$$

$$\phi_0 v_0 = c_1 v_0 + \sum_{p=2}^{\infty} p (c_1 v_0)^p e^{-(F_p - pF_1)} \tag{4}$$

The first equation is simply the law of mass action for the aggregate of size p. Using the notation $\Delta G_p \equiv F_p - pF_1 \equiv pg_p$, one can find the equilibrium partition of proteins among the different aggregates by solving the following non-linear equation in c_1

$$\phi_0 v_0 = c_1 v_0 + \sum_{p=2}^{\infty} p (c_1 v_0)^p e^{-pg_p} \tag{5}$$

and by plugging the solution into the law of mass action Eq.3. In order to address the question of dominance of a given population of particles with respect to another one, we restrict the model to a bimodal size distribution: the product of the self-assembly is either a small particle with p_1 proteins or a large particle with p_2 proteins. The equilibrium concentration of unaggregated proteins c_1 is now given by

$$\phi_0 v_0 = c_1 v_0 + p_1 (c_1 v_0)^{p_1} e^{-p_1 g_1} + p_2 (c_1 v_0)^{p_2} e^{-p_2 g_2} \tag{6}$$

The following argument shows that the influence of entropy within this equation is to favor the formation of smaller particles. Indeed after little algebra, it is possible to find an exact relationship between the concentration of initial protein ϕ_0 and the ratio between the equilibrium value of the number of particle 1 and 2 $\alpha = c_{p1}/c_{p2}$

$$\phi_0 v_0 = \left(\alpha e^{(p_1 g_1 - p_2 g_2)}\right)^{\frac{1}{p_1 - p_2}} + (p_1 + \frac{p_2}{\alpha}) \left(\alpha e^{(p_1 g_1 - p_2 g_2)}\right)^{\frac{p_1}{p_1 - p_2}} e^{-p_1 g_1} \tag{7}$$

In the case where the free energy of capsid formation is not size selective, one has $g_1 = g_2 \equiv g$, meaning that the energy per protein is the same in each particle irrespective of its size. This assumption is used in order to test the trend of the entropic contribution on the size distribution. Therefore the previous formula is simplified to

$$\phi_0 v_0 = \alpha^{\frac{p_1}{p_1 - p_2}} \left(\alpha^{\frac{1}{p_1}} e^g + \left(p_1 + \frac{p_2}{\alpha} \right) \right) \tag{8}$$

For $p1 < p2$, the ratio α is a decreasing function of the initial concentration ϕ_0. In other words, for initial concentration below a threshold value ϕ_{0*}, the small particles outnumber the large particles, while for larger concentrations the situation is reversed. The threshold concentration is given by

$$\phi_{0*} v_0 = e^g + p_1 + p_2 \tag{9}$$

Since for capsids the sizes are typically such that $p_1 >> 1$ and $p_2 >> 1$, the smaller capsids dominate on the whole concentration range.

In the opposite case where the free energy of capsid formation is size selective, meaning for example that $g_2 < g_1$ (all g's are negative for spontaneous self-assembly), the entropic effect favoring smaller particles has to be balanced with the enthalpic effect favoring larger particles. As a consequence, the maximal threshold concentration below which smaller particles dominate is decreasing as the enthalpic size selection favoring larger particles become more pronounced. The preference for smaller particles can eventually disappear for $g_2 << g_1$, meaning that entropic contribution is not able anymore to balance the enthalpic preference for large particles. The entropic selection of small particles is therefore subjected to an assumption of weak enthalpic size selectivity and disappears for strongly enthalpic size selection.

The previous reasoning can be generalized in order to describe the more complicated situation implying Gag proteins, viral RNA and cellular RNA. The main result of such a model is to predict that the entropy will contribute to two effects in a similar way to the situation with simple protein self-assembly: the first effect is an entropic selection of viral RNA against cellular RNA provided that its length is much longer to cellular RNA, and the second is the selection of smaller particles as in the previous case.

Focusing again on bimodal size distribution for the sake of clarity, we assume that the product of self-assembly is twofold: small particles (labeled "1") containing p_1 proteins, n_1 viral RNAs and m_1 cellular RNAs (which are assumed to be mono disperse in size), and large particles (labeled "2") containing p_2 proteins, n_2 viral RNAs and m_2 cellular RNAs. We further

assume that the total amount of nucleotides inside a particle is proportional to its number of proteins. As it is discussed in the main text of this work, this assumption has been verified on a large databases of RNA viruses. This assumption implies the following relation $p_{\{1,2\}} = K_v n_{\{1,2\}} + K_c m_{\{1,2\}}$, where K_v and K_c are respectively proportional to viral and cellular RNA length. The non linear equations to be solved in order to get the equilibrium partition of molecules within aggregates are now written as

$$\begin{aligned}
\phi_0 v_0 &= c_0 v_0 + p_1 c_{p_1} v_0 + p_2 c_{p_2} \\
\phi_{r+} v_0 &= c_{r+} v_0 + n_1 c_{p_1} v_0 + n_2 c_{p_2} \\
\phi_{r-} v_0 &= c_{r-} v_0 + m_1 c_{p_1} v_0 + m_2 c_{p_2} \\
c_{p_1} v_0 &= (c_0 v_0)^{p_1} (c_{r+} v_0)^{n_1} (c_{r-} v_0)^{m_1} e^{-p_1 g_1} \\
c_{p_2} v_0 &= (c_0 v_0)^{p_2} (c_{r+} v_0)^{n_2} (c_{r-} v_0)^{m_2} e^{-p_2 g_2}
\end{aligned}$$

These equations depend on a large set of parameters ($\phi_0, \phi_{r+}, \phi_{r-}, g_1, g_2, n_1,$ $n_2, m_1, m_2, K_v, K_c, v_0$), and so the general behavior is rich and complex. We have recently published a separate work dedicated to the detailed theoretical analysis of these equations (ref 28 fro main text). In particular, it is shown in this work that finer inclusion of polydispersity effects do change the qualitative picture drawn by the simple bimodal model. This justifies our use of this simpler approach. Numerical solutions for the equations of the bimodal models are presented in figure 5 and 6 of the main body of this work.
